# Practical and Theoretical Trials of “Formagen”

**Published:** 1898-06

**Authors:** Max Bauchwitz

**Affiliations:** Stettin, Prussia


### Abstracts and Translations.

### PRACTICAL AND THEORETICAL TRIALS OF "FORMAGEN."[1]

BY MAX BAUCHWITZ, STETTIN, PRUSSIA.

For more than eight months I have applied formagen in all stages of the inflammation of the pulp, and among over three hundred cases only two extractions were necessary afterwards. Unfortunately, I had the ill luck to receive in the first lot of formagen a preparation which was too powerfully saturated with the formaldehyde vapors, which naturally caused extremely severe pains, lasting for hours and days. Subsequent lots of formagen I received acted, however, almost painlessly; upon the application of the agent there followed only slight "dragging" in individual cases, periostitic irritation had to be recorded, which disappeared partly spontaneously and partly after treatment. Formagen has given splendid results in two of my own molars, one of which, after

[1] Translated from the Deutsche Monatschrift für Zahnheilkunde.

repeated but vain treatment, it had already been proposed to sacrifice. I have also to chronicle very good results in a series of pulpitic milk molars; it was applied in children at four years old, and has greatly contributed to the conservation of those teeth which otherwise would have fallen a prey to extraction, as the pulp-treatment with arsenic would in these cases have been out of place.

Thus it appears in formagen an agent has been found—*assuming that it is uniformly prepared, and the troublesome sticking of the cement on the instruments is overcome*—to which must be given the preference before many other medicaments for the treatment of pulpitis. For instance, a patient arrives with severe pulpitic pains; we excavate the carious tooth, the pulp is capped with the soft formagen cement, a filling is placed over it, and the patient, who was so fearful of the "nerve-killing," leaves the operating-room with only slight or mostly no pain at all. We, on the other hand, have the satisfaction of not having destroyed an organ as was done hitherto with arsenic, but, faithful to the principles of the healing art, to have preserved the organ which brings life to the tooth,—namely, the pulp,—with prospective *lasting* results. With this last I come to the question which every scientifically educated surgeon has a right to put to himself, but which up to the present has remained unanswered, *How does the formagen act upon the pulp?* Purely empirically, and, according to the expressed symptoms of the patients, which I am able to confirm by my experiences with my own teeth, formagen, which consists chiefly of carbolic-eugenol and an indifferent porous powder saturated with formaldehyde vapors, stills the pain almost instantly after the agent has been applied to the irritated pulp. Here it is that the well-known anæsthetizing action of the carbolic acid takes effect, which arises in this way, that it paralyzes the sensitive nerve-fibres of the pulp, and, furthermore, eugenol, the oxygenous constituent of oil of cloves, also acts as an anæsthetic. According to Liebreich, a local anæmia is not produced by the employment of eugenol, as with cocaine, but sometimes a painful sensation occurs, which the patient describes as "dragging"; this pain is connected with a rapidly transitory hyperæmia. The "dragging," indeed, is very soon felt on the application of formagen; it is, however, partly also to be attributed to the formaldehyde vapors, which develop rapidly and penetrate the pulp; and the more concentrated this penetration the more will the "dragging" rise to a real sensation of pain, as I will show later on. Under the filling which immediately follows, the pulp reacts normally and is sensitive to all injuries, whatever their nature may be, just as in healthy pulps. Now, in order to learn by means of theoretical and more instructive experiment the action of formagen and the changes produced by it in the tissue, I made a series of bacteriological investigations, such as were undertaken by Miller in his classical work, "Ueber die Mikroorganismen der Mundhöhle," on the pulp with the most varied antiseptics. For this purpose, like Miller, I chose the fleshy pulp of calves' teeth, which pulps, partly whole and partly split, I filled into a small tube which below ended in a pointed orifice. From this fine opening of the small tube, where mostly that part of the pulp came to lie which is situated at the apical foramen, I infected the pulp with bacteria of freshly extracted pulpitic teeth, and applied at the same time at the opposite end of the pulp—namely at its crown portion—the formagen, which I sealed with wadding and wax. The tube was placed with the infected part of the pulp into a reagent glass, which was partly filled with agar-agar culture, and was then deposited in the incubator at the temperature of the body. By this means I was able to observe from without the penetration of the formagen into the pulp, and thus also the process of putrefaction which might perhaps arise.

After one day the reddish pulp was converted on the surface into a solid, jelly-like structure, which on the second, third, and fourth day, and so on, became uniformly tough up to the extremest point. The peculiar pungent, formalin odor was completely maintained, and nowhere were processes of putrefaction to be observed. *Macroscopical and microscopical investigations demonstrated that no bacteria were present.* The macroscopical investigations were arranged in such a way that I transplanted the pulps, which were taken out of the small glasses, upon plates or saucers with agar-agar culture, and these again were deposited for various periods in the incubator. The plates remained completely sterile, *and hence is proved the extremely powerful disinfecting and antiseptic quality of formagen.*

Another question was, Does formagen act destructively upon the pulp-tissue, upon its nerves and blood-vessels, or does it conserve them? In order to answer this question, the pulps which had been treated in the manner mentioned above were fixed, and with the microtome sliced into extremely fine longitudinal and transverse sections. For confirmatory trials fresh calves' pulps were converted into preparations and examined microscopically like the former. The pulp treated with formagen showed, on the whole, the same picture as the normal ones. But with the former the blood was coagulated in all the vessels; the red blood-corpuscles, lying closely against each other, resembled a homogeneous brown-red pillar. The nerves appeared intact. As the blood-vessels had the same appearance also in the finest and most extreme ramifications of the pulp, it is to be assumed that the formaldehyde vapors set free had penetrated the entire pulp. Therefore the mode of action of the formagen would be as follows: On their application to the inflamed pulp the carbolic acid and eugenol act first anæsthetically; the latter a short time after produces a gentle dragging pain, by increasing slightly the hyperæmia present already with the inflammation. Shortly afterwards the formaldehyde vapors in the formagen stream gradually through the whole of the pulp tissue; they kill all pathogenic germs, and convert the pulp *in toto* into a jelly-like homogeneous mass, and stasis occurs. While stasis is naturally first formed in the blood-vessels lying nearest to the point of application of the formagen, these vessels receive new blood from below through the apical foramen, whereby the pressure in the vessels, narrowed through the stasis,—the vessels being full to bursting through the hyperæmia already existing in inflammation, and that called forth by the eugenol,—becomes so great that a portion of the sanguineous fluid is pressed through the vascular walls. This blood liquid is absorbed greedily by the porous powder contained in the formagen. By this means the vessels, which had severely pressed upon the nerves lying near them through their increased volume and thus caused pain, are relieved of the weight, a new circulation of the blood occurs, and in a short time after the pulp is in a condition to perform its function normally. A preparation, however, too strongly saturated with formaldehyde vapors acts too intensely and violently upon the blood-vessels, the pressure of the vessels, which have become suddenly and violently distended and burden the nerves, becomes over great, and thus perhaps is explained the severe pain, which I have often observed where the formagen has been too much saturated. On the basis of these experiments I have come to the conclusion that formagen can be recommended with a good conscience as a valuable agent closely approaching to Miller's ideal. It is not to be used in periostitis, as Abraham has pointed out already.

Finally, I should like to add here that my experiments have brought me to the conclusion that formagen does not influence plastic filling-materials prejudicially.—*Journal of the British Dental Association.*

